# Effect of transporting *Oreochromis niloticus* in water with and without sodium chloride on skin morphology and some immunity-related genes expression

**DOI:** 10.1186/s12917-024-03937-9

**Published:** 2024-03-16

**Authors:** Heba Naeim Sadek Hana, Mohamed Abd El Aziz Ahmed Abd El Galil, Mohamed Abd Allah Mousa, Rasha S. B. El-Lateif, Arafah M. Emam

**Affiliations:** 1 Unit of Fish Diseases, Assiut Lab, Animal Health Research Institute (AHRI), Assiut Lab., Assiut, Egypt; 2 Faculty of Veterinary Medicine, Sohag University, Sohag, Egypt; 3 National Institute of Oceanography and Fisheries, NIOF, Cairo, Egypt

**Keywords:** Oreochromis niloticus, Transportation, Stress, Gene expression, Mucin, Antimicrobial peptides, Defencin, Cathelicidin, Sodium chloride, SEM

## Abstract

The aim of this study was to examine the effects of salt addition on the skin gene expression of Mucin, Antimicrobial peptides, cortisol, and glucose in Oreochromis niloticus after 5-hour transportation in water. Three groups were compared: Control, post-transport without salt (PT-S), and post-transport with 5 g salt-1(PT + S), with a stocking density of 28.6 gL-1, 20 fish for each experimental group. The results showed that the PT-S group had more significant changes in gene expression than the PT + S group, suggesting that salt alleviated the stress and immune responses of O. niloticus. The PT-S group had higher expression of mucin- 2(MUC + 2) (7.58 folds) and mucin-5AC (MUC5-AC) (6.29 folds) than the PT + S group (3.30 folds and 4.16 folds, respectively). The PT-S group also had lower expression of β-defensin-1 (Dβ1) (0.42 folds), β-defensin-2 (Dβ2) (0.29 folds), and Cath1 (0.16 folds) than the PT + S group (0.82 folds, 0.69 folds, and 0.75 folds, respectively). The skin morphology of the PT-S group revealed some white patches with no goblet cell openings, while the PT + S group had better preservation of skin features with some goblet cell openings and slight white patches. This study indicates that O. niloticus can benefit from sodium chloride during transportation, as it helps to reduce stress and inflammation, balance mineral levels, enhance health and immunity, and regulate mucous secretion.

## Introduction

Nile tilapia (*Oreochromis niloticus*) is a valuable economic freshwater fish species and its performance and health condition can be negatively affected by stressful rearing conditions. One of the sources of stress for fish in aquaculture is the transportation of live fish, which involves mechanical and water quality deterioration stress on the fish [1]. To improve the profitability and sustainability of aquaculture, it is important to understand how commercial fish respond to stress [2]. Moreover, there is an increasing demand for objective criteria to evaluate the health and welfare of the fish [3]. The fish skin mucus is the viscous and slippery layer that covers the epithelial surfaces and protects the fish from pathogen invasion by providing a physical or chemical barrier [4]. During transportation, when fish are crowded in a single bag, they may lose more mucus, which compromises the mucus protective barrier and skin barrier homeostasis and may increase the susceptibility to diseases in a stressed fish.

Mucin and antimicrobial peptides (AMPs) are important components of fish skin mucus, which is a natural barrier that protects fish from various stressors, such as pathogens, parasites, predators, and environmental changes [5]. Fish transportation is a common practice in aquaculture and fisheries, but it can cause significant stress and damage to fish, leading to increased susceptibility to infections, reduced growth, and mortality [6].

Transporting fish in water can cause them to release cortisol, a hormone that indicates stress levels [7–9]. Previous studies have shown that stress protein genes are affected by stress and can serve as biomarkers for aquatic environmental quality [10, 11]. Therefore, it is important to reduce stress during transport [12]. A common practice in freshwater fish farming is to add salt (NaCl) to the water, which helps to prevent osmoregulation problems [13]. NaCl may also influence the immunity of fish skin, which is the focus of this work [14, 15]. We examined the expression of mucins genes (MUC2 - MUC5-AC), which are involved in mucus production, and antimicrobial peptide genes (Dβ-1, Dβ-2 and Cath-1), which are part of the innate immune system, in the skin of *Oreochromis niloticus* transported in water with or without salt. We also examined the impact of cytokines, prolactin and growth hormones on the sampe samples, and reported our findings in a separate publication [16].

## Materials and methods

### Fish transport experiments

Fish were obtained from Alaa Hussein Tilapia breeding farm at Assiut Province (Egypt). Fish were 100 ± 10 gm, fish were sampled before (control) and after a 5 h transport event. Transported groups consist of post transport fish in water without salt (PT-S) and post transport group in water with 5gm/L salt (PT + S). 5 g/L salt were chosen as our treatment based on our preliminary trials and the results of Oliveira et al. (2009) [17]. Transport water was directly obtained from the raft where the fish were held. Fish were not sedated during transport. 20 fish from each experimental group were sampled each time. Fish were anesthetized with MS-222 [18] before skin sampling. The fish was euthanized by spinal cord severance and skin dissected skin samples were preserved in RNA later and store at − 80 °C until analysis [19].

### RNA extraction and cDNA synthesis

RNA kit (Qiagen) was used to extract total RNA from the tissue samples (both challenged and healthy fish) following the manufacturer’s protocol. RNA purity and the concentration was determined at 260 /280 nm in nanodrop (Thermo Scientific). cDNA synthesis was carried out and examine by 1.5% agarose gel electrophoresis using the PrimeScript™ II 1st strand cDNA Synthesis Kit (TaKaRa, Japan) following the manufacturer’s protocol, using 2.5 mg of RNA as templates. The synthesized cDNA was then be diluted 40 fold in nuclease-free water and stored at 80- Cº for further use.

### Quantitative real time PCR (qPCR)

QRT-PCR was performed on the Roche Light Cycler 480 Real-time PCR System. The amplifications was performed in a total volume of 10 µl and included 5 µl of 2X SYBR Green Master Mix reagent, 1 µl of 1:10 diluted cDNA and 0.2 µl of each primer (10 µM). The thermal cycling profile consisted of an initial denaturation at 95 °C for 5 min followed by 40 cycles of denaturation at 95 °C for 15s, annealing at 60° for15s and extension at 72° for 20s. An additional temperature-ramping step from 95 °C to 65 °C will be used to produce the melting curve. The primer sets for quantification of mRNA levels of selected genes is designed based on the *O. niloticus* sequences found in Table 1.

**Table 1:** Oligonucleotide primers used in SYBR Green real time PCR

Gene	Primer sequence (5’-3’)	Reference
*EF-1α*	CCTTCAACGCTCAGGTCATC	Gröner et al., 2015 [35]
	TGTGGGCAGTGTGGCAATC	
*MUC-2*	CAACTGTTTTTGAGACAACTTCAGA	Midhun et al., 2019 [36]
	CTGAAGTGACCGTGGAAGG	
*MUC5-AC*	GCTCTGGTCTTCGGACTATCTG	Tacchi et al., 2015 [28]
	GCTGCTCTTACACAACGACG	
*DB-1*	GGTTTTCCTATTGCTTAATGTTGTGG	
	GACACACAGTTAAGTCATGG	
*DB-2*	GCTGACAGCAGTGCAAGCTGATGACAC	
	GCAAAGCACAGCATCTTAATCTGC	
*Cath-1*	ACCAGCTCCAAGTCAAGACTTTGAA	
	TGTCCGAATCTTCTGCTGCAA	

### Scanning electron microscopy

Tissue samples excised from fish of the groups I, II and III, were rinsed in physiological saline, dipped briefly in a 0.1% solution of S-carboxymethyl-L-cysteine (sigma Aldrich) to remove mucus following Whitear and Moate (1994) [20] and then fixed in cold (4 °C) 3% glutaraldehyde (Lifeline Medical, Inc) in 0.1 M sodium cacodylate buffer (pH 7.4) (sigma Aldrich) for 4 h. After fixation, the tissue samples were dehydrated, dried using Critical Point Dryer (E3000 series; Quorum Technologies), attached to stubs and then coated with gold using Sputter Coater (SC7620; Quorum Technologies) following Verma et.al.(2017) [21]. Processed samples were examined under a JEOL 5800LV scanning electron microscope.

### Statistical analysis

Results are expressed as the mean ± standard error (SE). Data analysis was performed in GraphPad Prism version 5.0 including normality tests. All data were normally distributed. Statistically significant differences were considered when *p* < 0.05. The qPCR measurements were analyzed by one and Two-way ANOVA test to identify statistically significant differences between groups. P value for each data analysis was recorded.

## Results

### Mucin genes expression

**Fig. 1:**
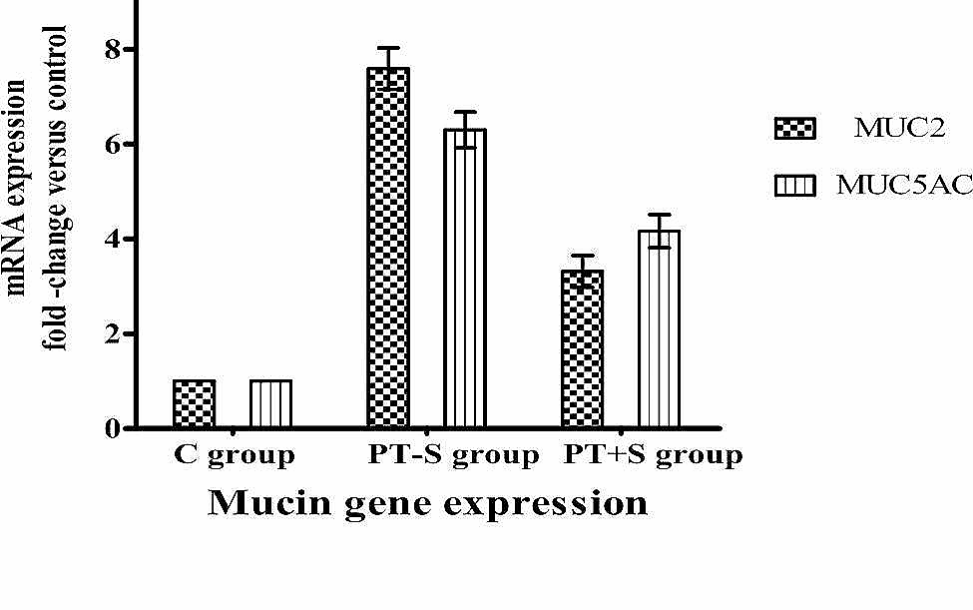
Mucins genes expression in skin of *O. niloticus* of PT-S and PT + S fish groups measured by RT-qPCR. Data are expressed as the mean fold-change compared to the control skin group. Bars represent means ± standard error. There was highly significant differences between groups according to Two-way ANOVA (*p* < 0.0001)

Mucin genes expression was significantly up-regulated in the PT-S and PT + S groups compared to the control group. *Mucin 2* (MUC + 2) gene expression was greater up-regulation in the PT-S group recording 7.58 folds comparing to a 3.30 folds in the PT + S group. Also, the *mucin 5AC* (MUC5-AC) gene expression was a greater up-regulate in the PT-S group and recorded 6.29 folds comparing to 4.16 folds in the PT + S group Fig. 1; Table 2.

**Table 2:** Mucin genes expression showed significant up regulation between groups and control according to Two-way ANOVA (*p* < 0.0001)

Fish group	Sample	Elongation factor-1 alpha (*EF1α*)	*MUC-2*		*MUC5-AC*	
		Cycle threshold (CT)	CT	Fold change	CT	Fold change
Pre-transport (CG)	A1	19.23	21.52	**-**	22.19	-
	A2	19.28	21.70		22.26	
	A3	19.38	21.85		22.44	
	Mean	19.30	21.69		22.30	
Post-transport in water without salt (PT-S)	B1	18.65	18.13	**7.52**	19.10	**5.86**
	B2	19.14	18.50	**8.17**	19.53	**6.11**
	B3	19.88	19.35	**7.57**	20.14	**6.68**
	B4	19.27	18.83	**7.11**	19.56	**6.54**
	Mean	19.24	18.70	**7.58**	19.58	**6.29**
Post-transport in water with 5 g/L salt (PT + S)	C1	19.15	19.90	**3.12**	20.15	**4.00**
	C2	20.47	21.04	**3.53**	21.25	**4.66**
	C3	18.29	19.12	**2.95**	19.24	**4.14**
	C4	21.11	21.63	**3.66**	22.16	**3.86**
	Mean	19.76	20.42	**3.30**	20.70	**4.16**

### Antimicrobial peptides genes expression

**Fig. 2:**
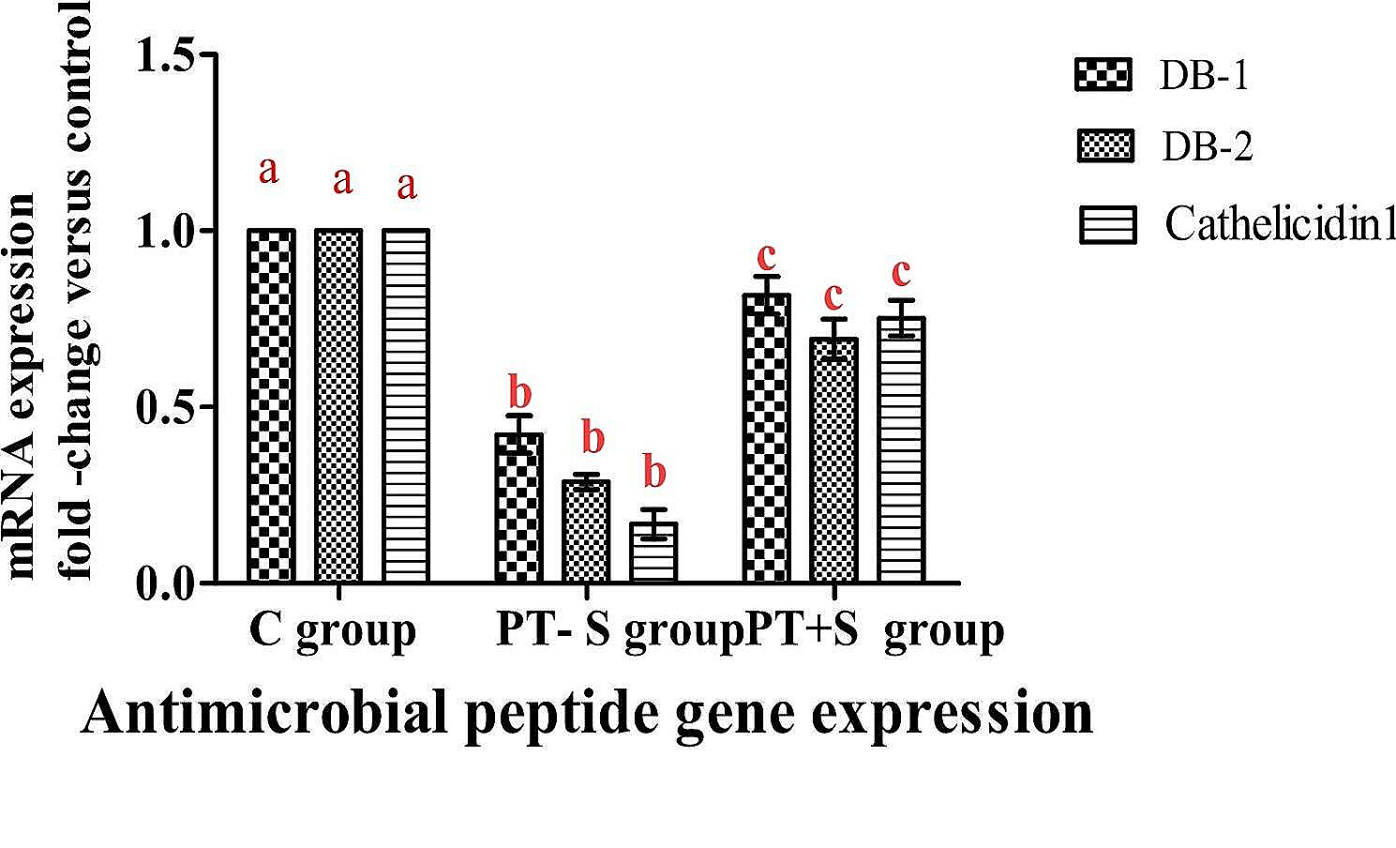
Reflected highly significant differences between antimicrobial peptide gene expression of the PT-S and PT + S groups compared to the control skin group. Bars represent means ± standard error. There was highly significant differences between groups according to Two-way ANOVA (*p* < 0.0001)

The evaluation of different antimicrobial peptides in response to the stress of transportation and the mitigation effect of Nacl revealed that *β-defensin-1* (Dβ-1), *β-defensin − 2* (Dβ-2) and *cathelicidin-1* (Cath-1) were significantly down regulated in the skin of fish of both post transport groups recording 0.42, 029 and 0.16 in PT-S group and 0.82, 0.69 and 0.75 in the PT + S group respectively. Overall, the down regulation of antimicrobial peptide genes was considerably more dramatic in the PT-S group than in the PT + S group Fig. 2; Table 3.

**Table 3:** Skin antimicrobial peptides genes expression showed significant down regulation between groups and control according to Two-way ANOVA (*p* < 0.0001)

Fish groups	Sample	*Elongation factor 1* *alpha (EF1α*)	Defensin- β 1 (*D* β *-1*)		Defensin- β 2 *(D* β *-2)*		*Cathelicidin 1* *(Cath 1)*	
		Cycle threshold (CT)	CT	Fold change	CT	Fold change	CT	Fold change
Pre-transport (CG)	A1	19.23	20.19	-	19.72	-	19.85	-
	A2	19.28	20.22		19.93		20.06	
	A3	19.38	20.30		20.03		20.20	
	Mean	19.30	20.24		19.89		20.04	
Post- transport in water without salt (PT-S)	B1	18.65	21.00	0.38	20.91	0.31	21.84	0.18
	B2	19.14	21.09	0.50	21.57	0.28	22.11	0.21
	B3	19.88	22.10	0.41	22.40	0.26	23.20	0.17
	B4	19.27	21.52	0.40	21.61	0.30	23.25	0.11
	Mean	19.24	21.43	0.42	21.62	0.29	22.60	0.16
Post- transport in water with 5 g/L salt (PT + S)	C1	19.15	20.34	0.84	20.19	0.73	20.28	0.76
	C2	20.47	21.60	0.88	21.48	0.75	21.66	0.73
	C3	18.29	19.57	0.79	19.48	0.66	19.31	0.82
	C4	21.11	22.45	0.76	22.36	0.63	22.37	0.70
	Mean	19.76	20.99	0.82	20.88	0.69	20.91	0.75

### Scanning electron microscopy (SEM)

**Fig. 3:**
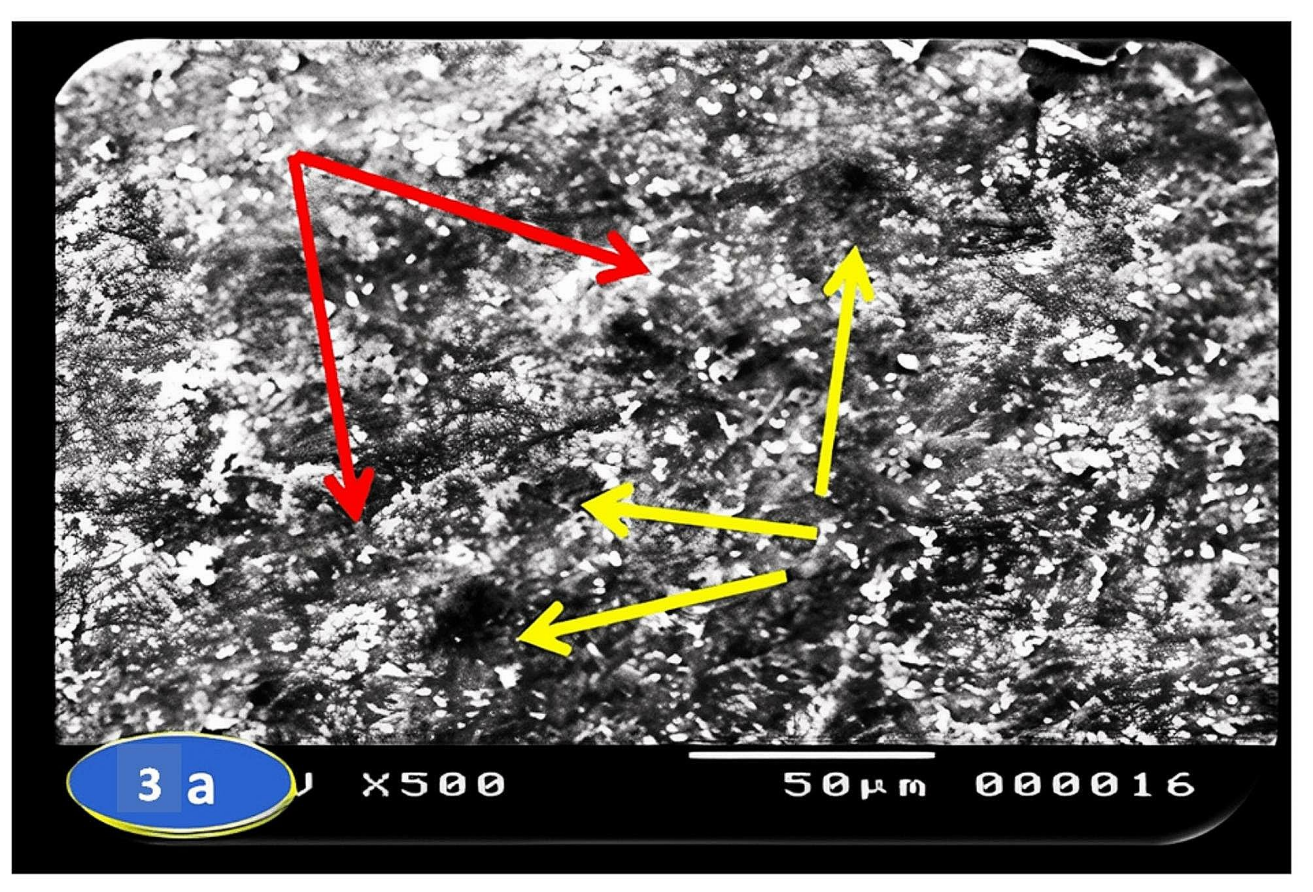
Scanning photographs for *O. niloticus* skin fish from control group taken at margination (x500 & x1000) showing goblet cells openings (yellow arrows) and the flat surface of squamous cells (red arrow)

**Fig. 4:**
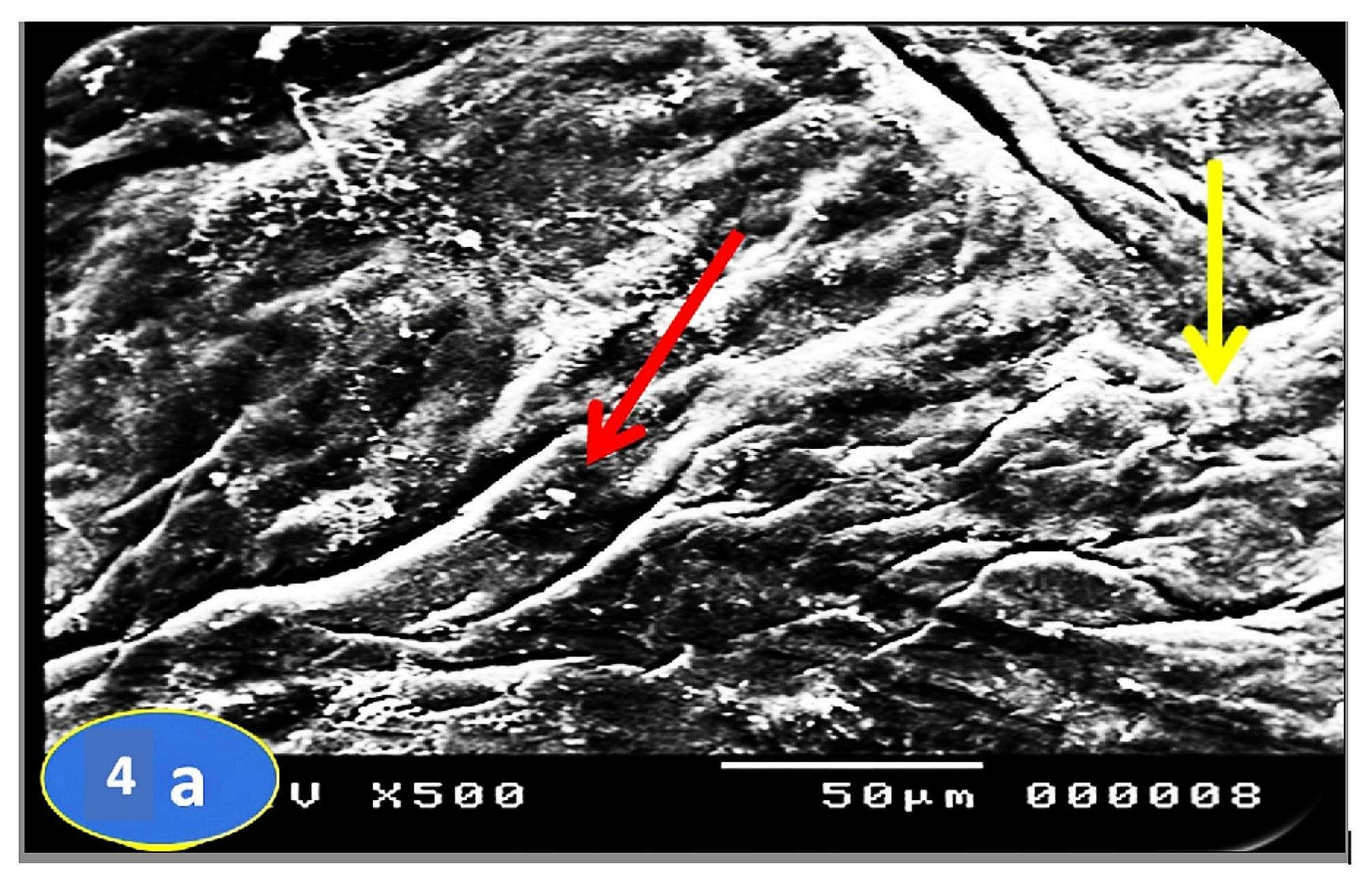
Scanning photographs for *O. niloticus* skin fish from PT-S group taken at margination (x500 & x1000) showing no or slightly clear goblet cells openings with cell debris (red arrow) and white patches may represent thickened surface (yellow arrow)

**Fig. 5:**
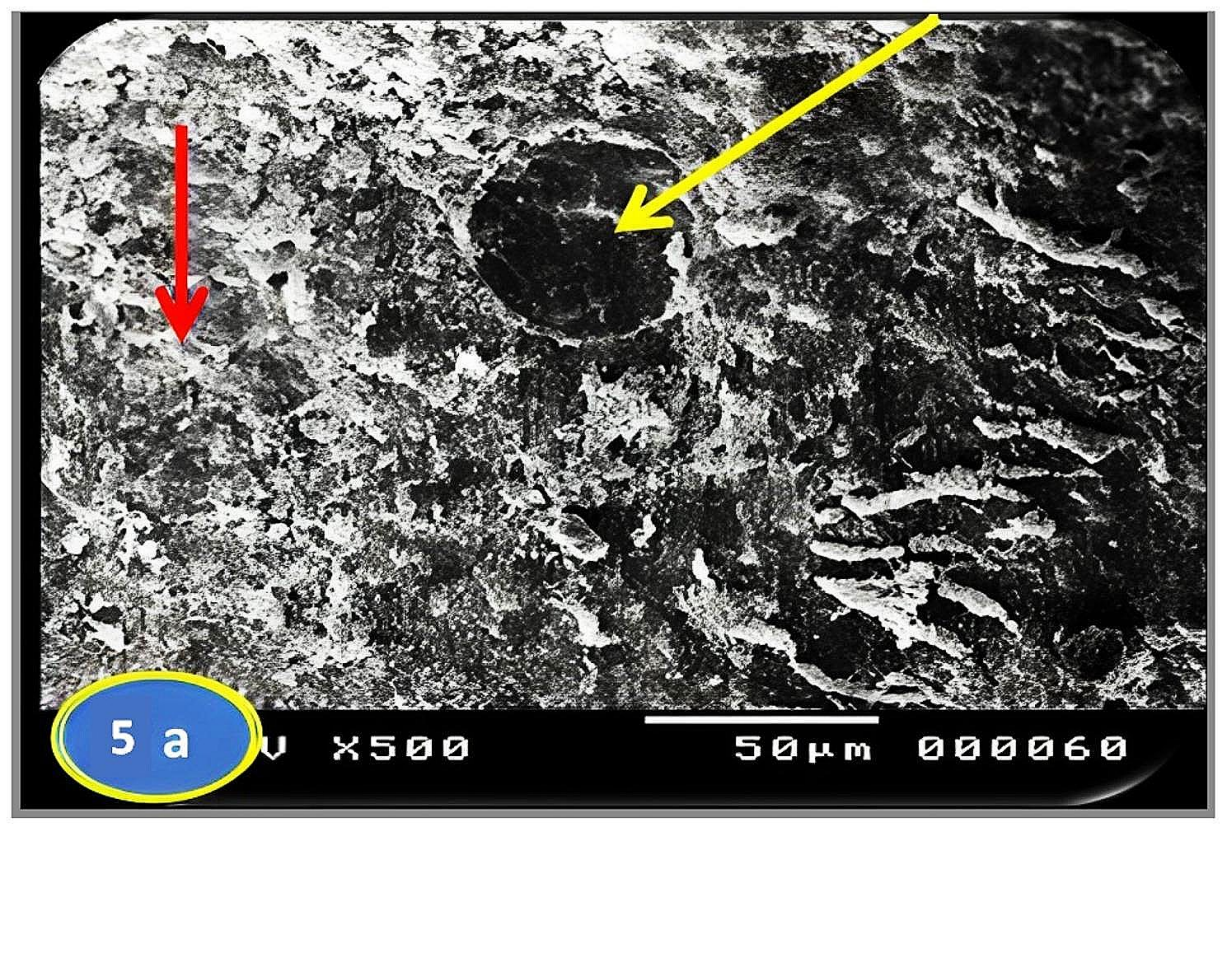
Scanning photographs for *O. niloticus* skin fish from PT + S group taken at margination (x500 & x1000) showing moderate preservation of surface skin features (red arrow) with goblet cells opening (yellow arrow)

Skin section of the control group fish showed the regular arranged pattern of surface squamous cells and minor depressions among superficial cells indicating opening of goblet cells (Fig. 3a & b). In a PT-S group, the skin showed scratched white patches that may represent thickened surface with absence or slightly clear goblets cell opening (Fig. 4a & b). On the other hand, the skin sections of the PT + S fish group showed moderate preservation of skin surface features with some goblet cells openings (Fig. 5a & b).

## Discussion

The transport of live fish is a common and inevitable practice in aquaculture [22, 23]. Transportation processes are stressful to fish [24] and the skin also expresses genes that may enhance immune system including antimicrobial peptides, cytokines, complements, major histocompatibility complex (MHC) and immune-globulins. These genes that are located in the skin produce substances which are then released to the surface and integrate with the mucus enhancing the first line defense in fish against pathogens [25]. The expression of the genes and immune parameters that were measured in this study could be helpful in monitoring the health status and welfare of *O. niloticus* particularly in relation to transportation stress.

Mucins are high molecular weight glycoproteins important for viscosity, trapping pathogens and physical barrier [26]. In this study, MUC + 2 and MUC5-AC genes expression were up regulated recording 7.58 and 6.29 folds in the skin of PT-S group and 3.30 and 4.16 folds in the skin of PT + S group comparing with the control group, their highest up regulation levels were detected in the skin of PT-S group. These findings clarified the fish skin secretes a large amount of mucins to increase the mucous defense function through trapping pathogen and physical barrier. The adding sodium chloride salt to the transport water of *O. niloticus* has a significant beneficial effect on mucous characteristics and mucin genes expression; these effects may come from the direct effect of water salinity. The upregulation of mucin gene expression in this investigation came in line with that of Ángeles (2012) [27] and Tacchi, et al. (2015) [28] reported increased production of cutaneous mucosal secretions in response to stress. Significant up-regulated of mucin genes were reported in the post transport fish by Tacchi et al. (2015) [28].

Antimicrobial peptides are a component of the innate immune system found in the surface layer of cytolytic and microbicidal epithelial tissues to inhibit the growth of bacteria, fungi, viruses, and parasites [29], they act as the first line of defense against various pathogenic microbial invasion without having high specificity or memory. Dβ-1, Dβ-2 and *Cathelicidin-1* are antimicrobial peptides significantly down-regulated in the skin of *O. niloticus* of PT-S and PT + S group. This downregulation of antimicrobial peptide genes was considerably more dramatic in the PT-S group indicating that transportation had an immune suppressive effect on the skin of *O. niloticus* In fact, antimicrobial peptide down-regulation in response to stress has been reported in fish by Noga et al. (2011) [30] and Tacchi et al. (2015) [28] whose found that a number of stresses lead to significant down regulation of AMPs. This downregulation was more dramatically in fish group transported in water without salt than in fish group transported in water with salt, the AMPs genes expression values were 7–17 times lower than controls [28]. The down regulation of AMPs genes expression in skin of PT + S *O. niloticus* group were lower than that of PT-S group indicating that the addition of salt to the transportation water had stress mitigation effect and alleviated the transport immune suppressive effect, this findings were supported by Mirghaed and Ghelichpour (2019) [31] who found that the addition of 3 g/L salt to transportation water seems to be beneficial for common carp as it mitigates water quality deterioration and immunosuppression. Expression of Dβ-1 and Dβ-2 in rainbow trout gills decreased after transportation stress, while Cath-1 expression in common carp skin increased. These findings indicate that the innate immune response of fish can be modulated by transportation stress through the expression of AMPs [32].

Scanning electron microscopy of skin revealed important differences between the *O. niloticus* groups. Skin section of the control group fish showed the regular arranged pattern of surface squamous cells and opening of goblet cells among superficial cells. In a PT-S group, scratched patches on the skin surface were reported and no goblet cells openings were observed on the external surface. The results of the PT-S group come in line with that of Ángeles (2012) [27] who reported that the stress changed the number of goblet cells and increased the amounts of mucus production in teleost skin, and Baldisserotto et al. (2007) [33] who reported disruption of epidermal mucus. The skin section of the PT + S fish group showed moderate preservation of surface skin features with goblet cells still open to the external surface and release their contents. These results suggest that salt has stress mitigation effect and may act as a regulator for the release of mucus from goblet cells in response to transport stress, also salt reduces the transport stress through reducing the need for energy for osmoregulation and maintenance of homeostasis. These results were supported with Nikinmaa, et al. (1983) [34] who suggested that salt in transport water reduce the difference between the fish internal osmolality and that of its environment, thereby reducing physiological workload required to maintain body homoeostasis.

Generally; the results of this investigation recorded distinguish anti-inflammatory effect in the skin of *O. niloticus* transported for 5 h in water without salt (PT-S group) coupled with up regulation in the MUC + 2 and MUC5-AC genes expression and higher down regulation in the antimicrobial peptides Dβ-1, Dβ-2 and Cathelicidin-1 genes expression comparing with their values in both *O. niloticus* control and the fish group transported in water with 5 g NaCl/L transport water (PT + S). Slight anti-inflammatory effect was recorded in the PT + S fish group comparing to the *O. niloticus* control group. These results revealed that *O. niloticus* in the PT-S group were exposed to higher transport stress effect and microbial invasion than the other two groups, and the addition of salt to the transport water mitigated the transport stress and reduce the chance of bacterial invasion through increasing the antimicrobial peptides production, enhancing mucous characteristics and restoring the mineral balance between fish and water and preserve the skin external surface. The complementary study showed that salt reduced inflammation in the skin and altered the expression of some genes and hormones. The salt group had higher levels of interleukin 1 and TGF-1a, which are involved in wound healing and tissue repair, and lower levels of prolactin and growth hormone, which are involved in cell growth and differentiation. The salt group also had higher cortisol levels, which is a stress hormone, than the control group and the post-transport group without salt [16].

## Conclusion

this study findings have significant importance to the field of fish aquaculture and underscore the importance of skin mucosal health during transportation, so that we recommend the using of sodium chloride during *O. niloticus* transportation, particularly when the fish are transported to long distance because the benefits of its using during transport seem to be reducing the stress inflammatory effects of transport on *O. niloticus* through restores the mineral balance between fish and water, improves the fish health and immune responses through increase the AMPs production and regulate the mucous release during transportation.

## Data Availability

All data generated or analysed during this study are included in this manuscript.
